# Handheld echocardiography in patients with cardiovascular disease: to use or not to use, that is the question

**DOI:** 10.1007/s12471-022-01741-4

**Published:** 2022-12-12

**Authors:** Eric Wierda, Berto J. Bouma, Renee B. A. van den Brink

**Affiliations:** 1 Department of Cardiology, Dijklander Hospital, Hoorn, The Netherlands; 2 Department of Cardiology, Amsterdam UMC, location AMC, Amsterdam, The Netherlands

**Keywords:** Handheld echocardiography, Physical examination, Handheld ultrasound-assisted physical examination, Coronavirus disease 2019

## Abstract

The physical examination is one of the most important diagnostic tools for physicians. Traditionally, a physical examination consists of inspection (looking), palpation (feeling), percussion (reflection of sound) and auscultation (listening). Handheld echography devices could become the new fifth element of a physical examination. The use of handheld echocardiography has recently increased because the devices have become smaller, easier to handle and more affordable. Handheld echocardiography is used by many specialists involved in acute cardiovascular care. In this narrative review we give a summary of the diagnostic accuracy and limitations of cardiovascular physical examination combined with handheld echocardiography. In patients with cardiovascular disease, adding handheld echocardiography to physical examination increases the sensitivity for detecting valvular heart disease (71% vs 46%) and left ventricular dysfunction with an ejection fraction < 50% (84% vs 43%). Handheld echocardiography might be better for ruling out diseases with a low pre-test probability than in confirming diseases with a high pre-test probability.

## Introduction

The physical examination of a patient has been an important diagnostic tool for physicians for ages. As long ago as 400 years B.C., Hippocrates of Cos described the value of clinical perception by “sight, touch, hearing, smell, taste and understanding” [1]. The advantages of a physical examination are: it can always be performed, it is safe and mostly non-invasive. Moreover, physical examination can enhance the physician-patient relationship: patients expect some form of physical examination as part of the evaluation when visiting a physician [2].

There has always been tension between physical examination and technological developments. When Laennec—the inventor of the stethoscope—wrote in 1819 that every case of pneumonia could be detected by lung auscultation, other physicians questioned the diagnostic accuracy of this new technique [3]. New developments in cardiovascular imaging augment this tension and favour increased dependency on imaging rather than physical examination. Other factors that hinder the performance of a physical examination are inadequate training of essential skills and time restrictions.

One of the recent technological developments in cardiovascular imaging is the miniaturisation of echocardiography devices. Traditionally, a physical examination includes inspection (looking), palpation (feeling), percussion (reflection of sound) and auscultation (listening). In the recent literature, handheld echography is considered the new fifth tool in addition to the traditional parts of a physical examination [1]. Recently, during the coronavirus disease 2019 (COVID-19) pandemic, lung ultrasound has proven to be extremely helpful in diagnosing and monitoring patients with COVID-19 [4].

In this narrative review, we examine the possibilities and pitfalls of handheld echocardiography and handheld ultrasound-assisted physical examination (HUAPE) in patients with cardiovascular disease. We describe the diagnostic accuracy of the traditional physical examination and the diagnostic accuracy of physical examination including handheld echocardiography. Finally, we make recommendations on how to regulate competence in handheld echocardiography for different specialties involved in acute cardiovascular care.

### The diagnostic accuracy of cardiovascular physical examination

How accurate is physical examination of the cardiovascular system by a skilled physician in making a correct diagnosis? In the literature, most often the diagnostic value of a physical sign is judged on its own merits and compared with a reference standard (often echocardiography), while in clinical practice a combination of clinical history (symptoms) and multiple signs on physical examination is used to make a diagnosis. The accuracy of a physical sign is described using sensitivity, specificity and likelihood ratio (LR) [5]. The LR of a physical sign is the probability of finding the sign in patients with a disease, divided by the probability of finding the sign in patients without a disease [6].

**Fig. 1:**
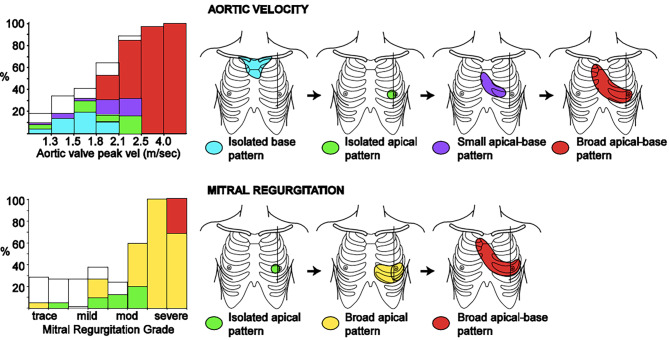
Murmur pattern of isolated aortic stenosis (*n* = 247) and mitral regurgitation (*n* = 174). The *vertical axis* represents the percentage of patients who have the murmur pattern on auscultation. The *horizontal axis* shows the echocardiographic severity of the valvular lesion. Peak velocity of 4 m/s indicates severe aortic stenosis. *vel* velocity, *mod* moderate. (Reproduced from [7] with the permission of *The American Journal of Medicine*)

To what extent a physical sign argues in favour of a disease depends on the pre-test probability of that disease. Rather complicated calculations have to be made using the pre-test odds (conversion of probability to a number), multiplying this by the LR to find the post-test odds, which should then be converted back to post-test probability (a percentage). However, it is also possible to use a table with good matching estimates. For example, an LR of more than 5 indicates strong diagnostic evidence and increases the pre-test probability by around 30% [6]. Tab. 1 shows physical signs in severe aortic stenosis and mitral regurgitation with a high LR (and their corresponding approximate changes in pre-test probability) [7]. Fig. 1 shows distribution patterns of systolic murmurs in these valvular lesions [7].

**Table 1:** Physical signs of severe valvular heart disease with high likelihood ratios (LRs) and their corresponding approximate change in probability

Physical signs of severe valvular heart disease	LR and change in pre-test probability
*Aortic stenosis*	
Delayed upstroke carotid pulse	LR + 6.8 (+35–40%)
Diminished or absent S2	LR + 7.5 (+35–40%)
Carotid radiation	LR + 8.1 (+40%)
Systolic murmur with broad apical-base pattern (see Fig. 1)	LR + 9.7 (+45%)
*Mitral regurgitation*	
Systolic murmur with broad apical pattern (see Fig. 1)	LR + 6.8 (+35–40%)
Systolic murmur with loud S2	LR + 4.7 (+30%)

*S2* second heart sound

Limitations of physical examination include poor standardisation of skills and insufficient training. Mastering the physical examination has nothing to do with innate talent, but much more with continued practice. The old saying “See one, do one, teach one” should in this case be adjusted to “Taught well, repeat, repeat, repeat” [8]. Medical students and residents should be taught valuable physical signs with either very high or very low LRs, which either significantly increase (LR more than 5) or decrease (LR less than 0.5) the probability of common cardiovascular diseases [3]. Education and continued practice of cardiovascular physical examination should include both bedside teaching by skilled physicians as well as learning by using training simulators. Skills like correct placement of the stethoscope on the chest, auscultation of murmurs, rubs and extra heart sounds, assessment of the jugular venous pressure, tactile resemblance of pulses, location of apical beat and measurement of blood pressure should not only be taught but also practised [9]. High-quality, online-accessible training courses can improve auscultation skills [10–12]. In many medical schools testing of skills required for a physical examination is done by objective structured clinical examination [13].

### The diagnostic accuracy of handheld echocardiography

**Fig. 2:**
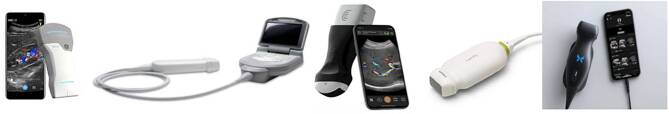
Currently available handheld ultrasound systems for performing echocardiography. From *left* to *right*: Vscan (GE Healthcare), Acuson P10 (Siemens), PA HD (Clarius), Lumify S4‑1 (Philips), iQ (Butterfly)

A promising approach for improving the diagnostic accuracy of cardiovascular physical examination is the addition of bedside handheld ultrasound. Portable ultrasound devices can be subdivided into three groups: laptop-associated devices, hand-carried units and handheld systems. The use of handheld echocardiography devices has increased recently because these devices have become smaller, easier to handle and more affordable. Tab. 2 and Fig. 2 show an overview of the currently available devices. Overall, the quality of handheld echocardiography is lower and the possibilities of such devices are fewer than those of fully equipped high-end echocardiography machines. Handheld echocardiography devices support two-dimensional echocardiography and colour Doppler. However, most devices have no spectral Doppler option and all devices lack simultaneous electrocardiogram tracing. Storage issues are common in daily practice. Due to the integration of colour Doppler, these devices are suitable for detecting regurgitant lesions and to some extent capable of differentiating mild from severe regurgitation. However, because spectral Doppler is frequently lacking, handheld devices cannot always be used to assess the severity of stenotic lesions, pulmonary artery pressure or diastolic left ventricular (LV) function.

**Table 2:** Currently available handheld ultrasound systems for performing echocardiography

Type of device	Company	Weight	Display^a^	2D Doppler	MHz	Colour Doppler	CW or PW Doppler	Estimated price
Vscan	GE Healthcare	450 g	Yes	Yes	3–8	Yes	No	5300 €
Acuson P10	Siemens	700 g	Yes	Yes	2–4	No	No	3600 €
PA HD	Clarius	350 g	No	Yes	1–4	Yes	Yes	4400 €
Lumify S4‑1	Philips	150 g	No	Yes	1–4	Yes	No	6500 €
iQ	Butterfly	300 g	No	Yes		Yes	No	1899 €

*CW* continuous wave, *PW* pulsed wave

^a^Not including display = probe needs to be connected to external (handheld) device

The advantage of handheld echocardiography is that the examination can be performed quickly (< 5 min); it can provide answers to specific questions (see Tab. 3) in symptomatic patients or detect abnormalities in asymptomatic patients. In a systematic review it was found that, compared with traditional physical examination, HUAPE increased the sensitivity for detecting aortic or mitral valve disease (71% vs 46%) and LV dysfunction with an ejection fraction < 50% (84% vs 43%), but specificity was equal in aortic or mitral valve disease (94% vs 94%) and did not differ significantly in LV dysfunction (81% vs 89%). Handheld echocardiography might be better for ruling out diseases with a low pre-test probability than in confirming diseases with a high pre-test probability [14]. In addition to benefits related to diagnostic accuracy, time reduction in making a diagnosis and improved patient satisfaction could also be advantages. It is important to take into account that in the studies included in the review, clinical setting, level of training and skills and standardisation of clinical assessment (especially for LV dysfunction) differed importantly.

**Table 3:** Relevant targets for physical examination combined with handheld echocardiography

Relevant target with handheld echocardiography	Parameter
Left ventricular systolic function and dimensions	Visual estimation of left ventricular ejection fraction, possibility of post-processing of images to calculate ejection fraction
Right ventricular systolic function and dimensions	Visual estimation of right ventricular function and dimensions
Left and right atrial sizes	Left atrial diameter
Valvular heart disease	Morphological valvular abnormalities
Large intracardiac masses	
Pleura and lung parenchyma	B‑lines in pulmonary congestion Pleural effusion
Intravascular volume assessment and screening for aortic aneurysm	Intravascular volume assessment (inferior vena cava diameter), diameter of thoracic and abdominal vessels
Pericardial effusion/tamponade	Amount of pericardial effusion

**Fig. 3:**
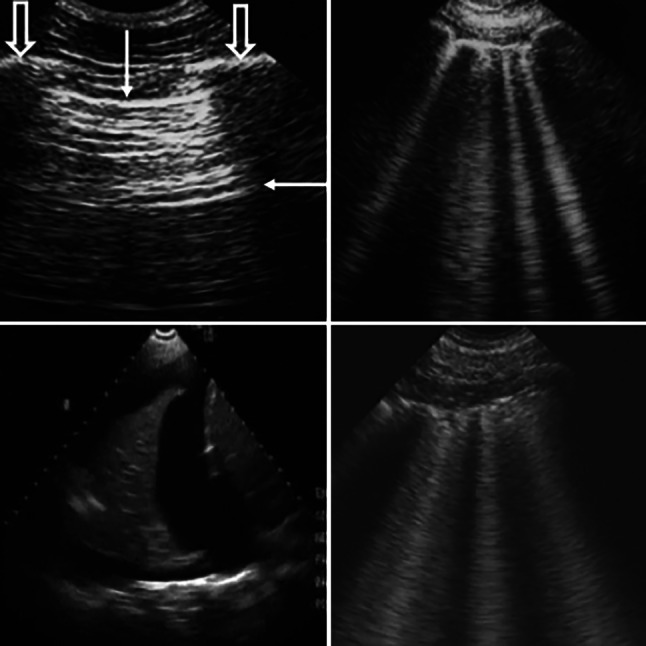
Lung ultrasound. *Upper left* Normal pattern (“dry lung”). Note the thin clear pleural line (*downwards arrow*) and two ribs with acoustic shadowing (*open downwards* *arrows*); note also the A‑line (*leftwards arrow*, repetition artefacts of the pleural line). *Upper right* Pulmonary congestion with a normal pleural line and 4 B-lines (like comet tails replacing the normal A‑lines). *Below left* Pleural effusion and consolidation. *Below righ*t The pleural line is irregular and thickened, usually indicating pneumonia and/or acute respiratory distress syndrome. Reproduced from [24] with the permission of *JACC Cardiovascular Imaging*

Handheld devices could also be used for purposes other than echocardiography, for example to perform lung ultrasound or for vascular access. In cardiovascular examinations, handheld devices could be used to assess the cause of dyspnoea (by looking for B‑lines, pleural effusion, atelectasis or consolidation) and to improve the accuracy of diagnosing acute heart failure [15]. The so-called “B-profile” (combination of more than three B‑lines in two intercostal spaces bilaterally and normal lung sliding) identifies a cardiogenic origin of dyspnoea with a high sensitivity and specificity (94% and 92%, respectively) [16, 17]. Currently, lung ultrasound is used in diagnosing and monitoring patients with COVID-19 and has been shown to be more sensitive than the traditional chest X‑ray [18]. Lung ultrasound has also been shown to have a good correlation with computed tomography findings. See Fig. 3 for examples of images obtained by lung ultrasound.

### Training and testing of competence in handheld echocardiography

There is a risk that handheld echocardiography devices might be inappropriately used by undertrained individuals [19]. The amount of training required for physicians in the acquisition and interpretation of ultrasound images depends on their background knowledge and echocardiography skills. Residents in cardiology already receive comprehensive training in echocardiography in their teaching hospital. In some European countries, residents participate in the European transthoracic echocardiography examination and certification programme [20]. In addition, the European Association for Cardiovascular Imaging (EACVI, part of the European Society of Cardiology) offers an online education and certification programme for handheld echocardiography [21]. To improve knowledge and proficiency, additional training in handheld echocardiography should be incorporated in imaging internships. Also, the European transthoracic echocardiography examination and certification programme should include questions about handheld echocardiography and lung ultrasound.

With regard to non-cardiologists, in 2018 the EACVI issued a consensus statement, with the intention of ensuring the accuracy and quality of focused cardiac ultrasound in emergency settings by non-cardiologists [22]. Minimal education and training requirements for achieving competence in performing handheld echocardiography are described, including a basic theoretical knowledge of cardiovascular disease, review of pre-recorded cases, and guidance on mastering the technique. The EACVI, however, places the responsibility for implementing training programmes on the different professional organisations (emergency physicians, intensive care specialists, pulmonologists, cardiac surgeons and others). Specifically for lung ultrasound, it is advised that a physician should perform a total of 25 supervised examinations before being considered competent [23]. In daily practice, however, the usage and amount of training in specialties other than cardiology is very diverse and the use of handheld devices without appropriate skills is certainly conceivable. Because there are no binding European or national guidelines, it is important for cardiologists to make local agreements with other physicians performing handheld echocardiography as regards training, implementation and storage of images.

### Final remarks

Handheld echocardiography is a promising approach for improving the diagnostic accuracy of cardiovascular physical examination. Handheld devices will most probably become a part of daily clinical practice in the future. We feel it is a valuable addition to (but not a replacement for) the physical examination. It is important to realise that handheld echocardiography is not meant—and not able—to replace clinical judgement, physical examination and full echocardiographic examination. The results of handheld echocardiography should always be combined with the findings of physical examination and must be interpreted with the probability of disease in mind.
